# Recurrent retroperitoneal liposarcoma with multiple surgeries: a case report

**DOI:** 10.3389/fonc.2024.1363055

**Published:** 2024-05-03

**Authors:** Xiao Wang, Xiaobiao Song, Qiang Song, Jijun Wang, Junsheng Chen

**Affiliations:** ^1^ Department of Gastrointestinal Surgery, Baotou Central Hospital, Baotou, Inner Mongolia, China; ^2^ Baotou Clinical Medical College, Inner Mongolia Medical University, Baotou, Inner Mongolia, China

**Keywords:** retroperitoneal liposarcoma, recurrence, surgical treatment, auxiliary treatment, case report

## Abstract

Retroperitoneal liposarcoma (RPLPS) is a rare malignant tumor that is typically treated with surgical resection. However, RPLPS often has a high rate of local recurrence, making it crucial to explore new treatment options. In this report, we present the case of a middle-aged woman who experienced seven recurrences and underwent seven surgeries following the initial resection. Currently, the patient’s condition remains stable after the eighth surgery. Although there have been numerous reports of RPLPS cases both domestically and internationally, instances of repeated recurrence like this are exceptionally rare. Therefore, we have gathered the patient’s case data and conducted a retrospective analysis, incorporating relevant literature, to enhance the understanding of this disease among clinical practitioners.

## 1 Introduction

Liposarcoma (LPS) is the most common subtype of soft tissue sarcomas (STSs), accounting for 20% of all STSs (1). Pathologically, LPS is divided into five types (2): well-differentiated liposarcoma (WDLPS), dedifferentiated liposarcoma (DDLPS), myxoid liposarcoma (MLS), pleomorphic liposarcoma (PLPS), and myxoid pleomorphic liposarcoma (MPLPS). LPS originates from primitive mesenchymal cells differentiated from adipocytes (3) and is most commonly found in the extremities (52%) and retroperitoneum (13%) (4). It is worth noting that due to the large retroperitoneal space, retroperitoneal liposarcoma (RPLPS) can often grow to extremely large sizes. Consequently, RPLPS is typically asymptomatic in the early stages until the tumor enlarges and compresses surrounding organs, leading to noticeable symptoms (5). This characteristic makes early diagnosis and subsequent effective treatment challenging. Currently, surgical resection is the primary treatment method for RPLPS (6). However, even after successful tumor resection, most patients still require additional treatment modalities due to the higher recurrence rate of RPLPS compared to LPS in other locations. These additional treatment modalities may include surgery, radiotherapy, chemotherapy, or targeted therapy (7, 8). In this study, we present a case of RPLPS with repeated recurrence and multiple surgeries, and provide a comprehensive overview of the current treatment methods for RPLPS.

## 2 Case description

The patient, a 37-year-old female, presented to our hospital on January 9, 2017 with a history of retroperitoneal tumor resection 8 months prior. She had noticed an abdominal mass for the past month. The initial tumor resection had taken place at the Retroperitoneal Tumor Surgery Department of the People’s Liberation Army General Hospital in Beijing in April 2016. The tumor weighed approximately 4.6kg and was diagnosed as liposarcoma based on the postoperative pathology report. One month before her current visit, the patient discovered a palpable mass on the right side of her abdomen, along with a mild bloating sensation. The patient reported no prior instances of hypertension, diabetes mellitus, coronary heart disease, or any allergies to drugs or food. During the physical examination, a flat abdomen was observed along with a scar from a previous surgical incision in the upper abdomen’s center. Additionally, a hard, irregular mass was identified on the right side of the abdomen. An enhanced CT scan of the abdomen revealed a space-occupying lesion measuring 9.3×6.4×11.3cm in the right abdominal cavity. It also showed slight dilation of the right renal pelvis and compression of the right ureter. Based on the patient’s medical history, physical examination, and CT findings, the clinical team diagnosed the mass as recurrent retroperitoneal tumor. On January 16, 2017, the patient underwent right retroperitoneal tumor resection and right hemicolectomy. The size of the resected tumor was approximately 20×15×15cm. The postoperative pathological diagnosis confirmed the presence of retroperitoneal dedifferentiated liposarcoma, localized myxoid liposarcoma, and involvement of the mesentery, right renal fat sac, and adrenal nodular hyperplasia. There was no involvement of the omentum or appendix. The stump and periintestinal lymph nodes showed no evidence of tumor spread with 0/9 lymph nodes affected. As the surgical resection was deemed complete, the patient did not receive postoperative radiotherapy or chemotherapy.

**Figure 1:**
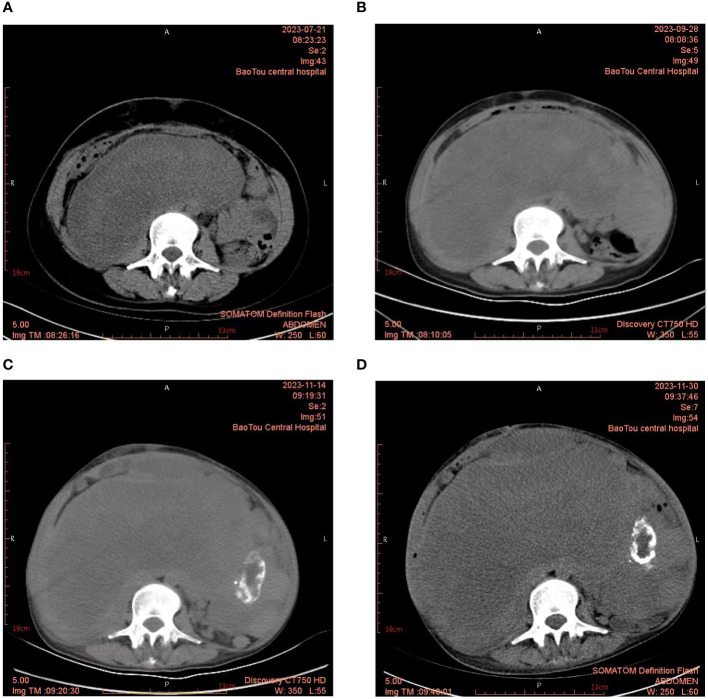
**(A–D)** Abdominal CT showed multiple huge masses in the abdominal cavity. By the eighth surgery, the larger masses had grown to 23×12 cm.

**Figure 2:**
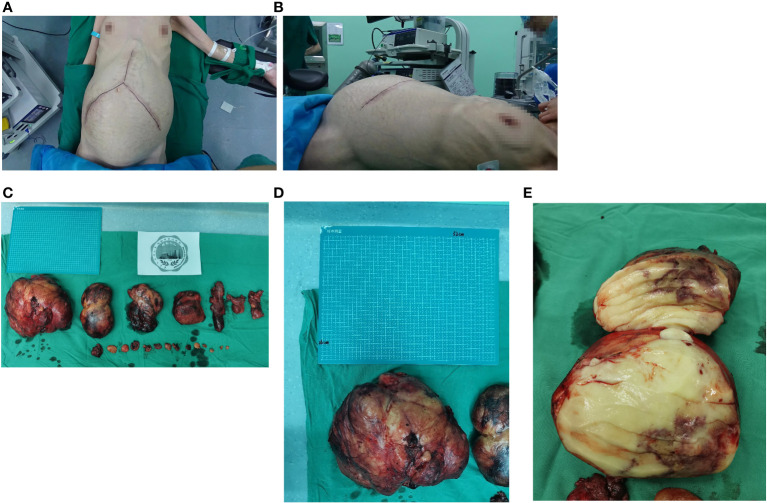
**(A, B)** Frontal and lateral appearance of the patient’s abdomen before the eighth surgery. **(C)** A total of 21 retroperitoneal tumors were removed in the eighth operation. **(D, E)** The maximum size of the tumor is 32×26cm, and the cut surface is fish-shaped.

**Figure 3:**
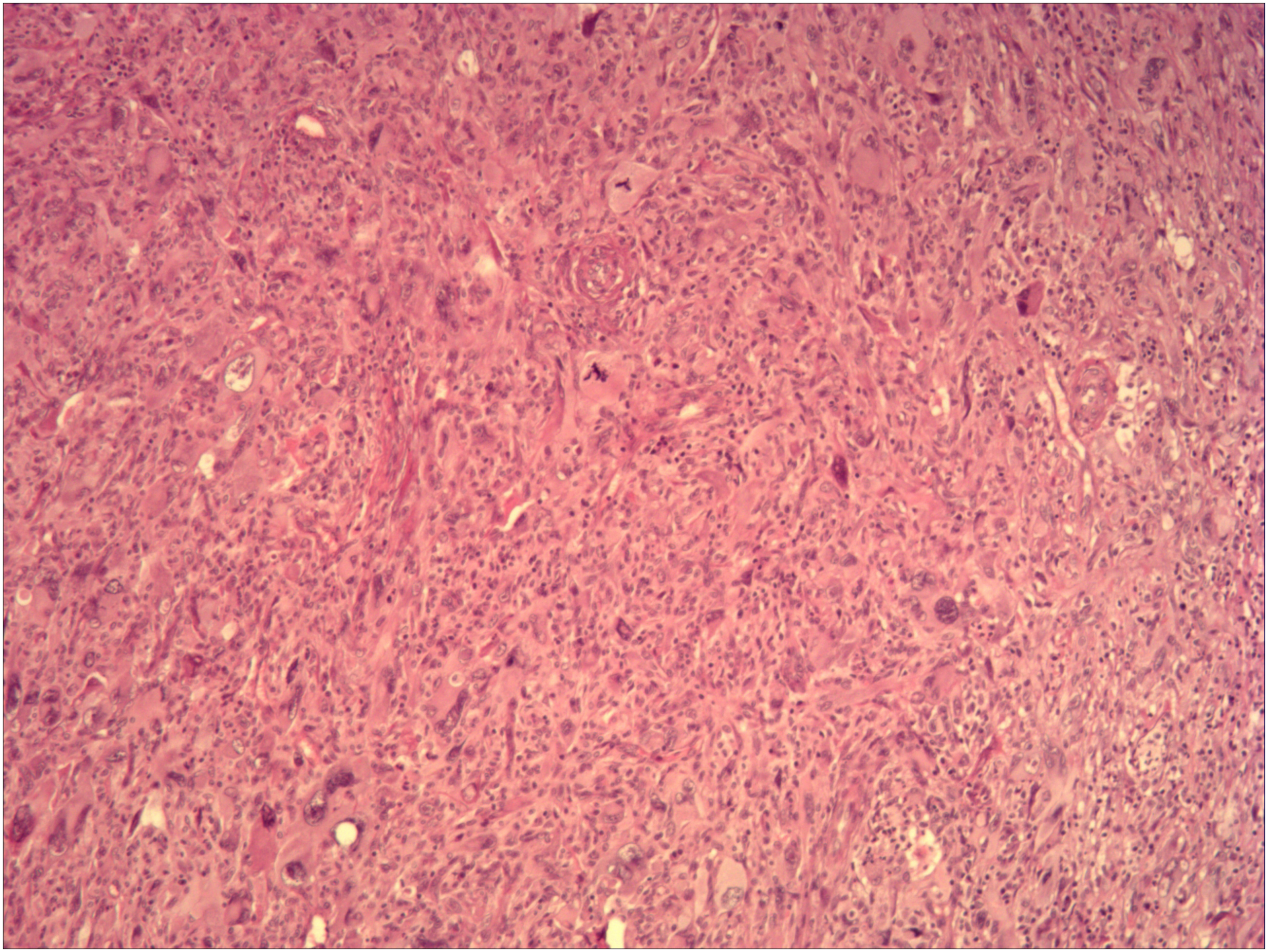
The patient’s eighth postoperative pathological analysis (hematoxylin and eosin staining, ×100 magnification) showed dedifferentiated liposarcoma, with localized pleomorphic liposarcoma.

**Figure 4:**
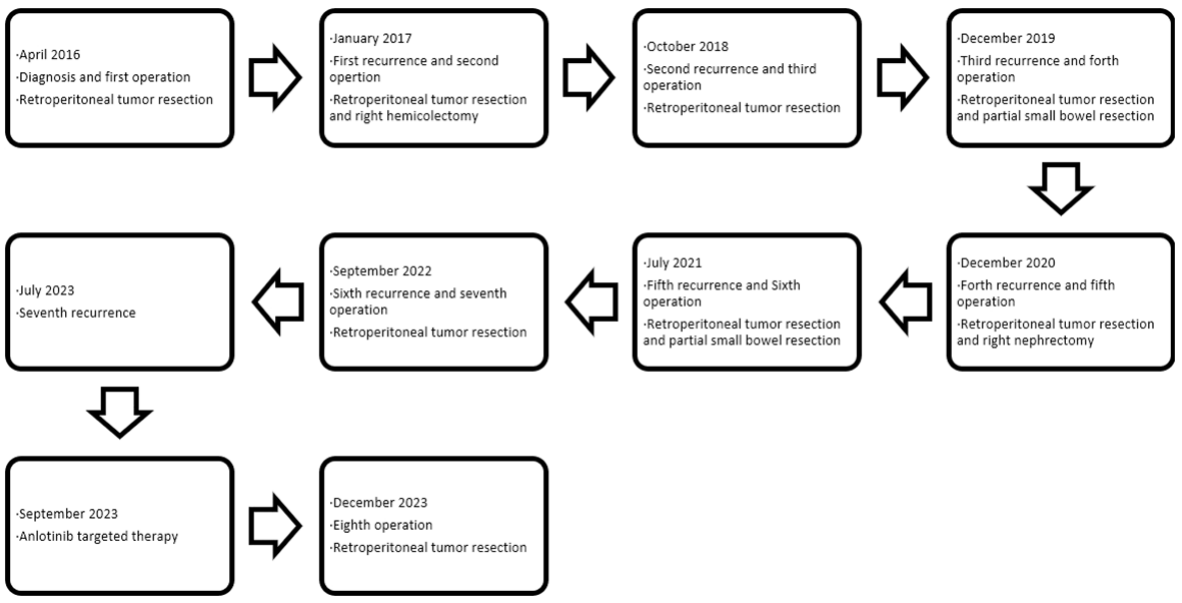
Timeline of this case.

The patient was regularly followed up after surgery until the local recurrence of the tumor was discovered on October 19, 2018. Subsequently, the patient’s RPLPS has relapsed multiple times on the following dates: October 30, 2018; December 31, 2019; December 5, 2020; July 31, 2021; September 22, 2022; and December 14, 2023. Tumor resection was performed through open surgery. In January 2020, the patient underwent a comprehensive gene test, which revealed an insertion-deletion mutation in the patient’s somatic KMT2D gene, with a mutation frequency of 1.3%. Chemotherapy was initially considered for the patient, however, their financial constraints posed a challenge in affording long-term treatment. Furthermore, due to the frequent tumor recurrences and the limited interval between them, it was uncertain whether chemotherapy would yield the desired outcomes. Consequently, after thorough deliberation, the patient decided to forgo the treatment plan. Despite undergoing several courses of anlotinib targeted therapy during the patient’s seventh relapse, there was no significant improvement in their condition. Throughout the course of the disease, the patient has experienced a total of 7 recurrences and has undergone 8 surgeries. **Figure 1** displays the abdominal CT scan since the seventh recurrence, illustrating the presence of multiple tumors. The eighth operation revealed the largest tumor measuring 32 × 26 cm, with a total weight of 12 kg (**Figure 2**). During the second surgery, the patient underwent a right hemicolectomy due to colon involvement, and in the fifth surgery, the right kidney was removed due to tumor invasion into the right renal parenchyma. All postoperative pathological diagnoses primarily indicated DDLPS, with local WDLPS, MLS, and PLPS also present (**Figure 3**). The timeline of this case is depicted in **Figure 4**.

## 3 Discussion

RPLPS is a rare mesenchymal tumor, accounting for approximately 0.07% to 0.2% of all tumors (9). It typically affects individuals aged 40 to 60 years, with a relatively equal gender distribution (10). The American Cancer Society (ASC) has identified several risk factors for LPS, including radiation (especially radiation therapy for other malignancies), certain familial cancer syndromes, lymphatic system damage or trauma, and exposure to toxic chemicals (11). According to the classification of STSs by the World Health Organization, the subtypes of LPS include WDLPS, DDLPS,MLPS, PLPS, and MPLPS (2). Among these subtypes, PLPS and MLPS are more commonly found in the extremities, while WDLPS and DDLPS are more commonly found in the retroperitoneum (12).

The clinical manifestations of early RPLPS are usually not significant and are often detected at an advanced stage, characterized by a large abdominal mass (13). Many patients do not experience any symptoms, but if present, they may include nonspecific symptoms like flank pain, early satiety, or general discomfort (14). In this case, the patient did not exhibit any obvious physical signs initially, but a palpable abdominal mass was identified.

Computed Tomography (CT) is widely used for the diagnosis and preoperative evaluation of Retroperitoneal Liposarcoma (RPLPS) (15). However, Magnetic Resonance Imaging (MRI) offers higher resolution of soft tissues, enabling more accurate diagnosis of retroperitoneal tumors. MRI also provides clear visualization of tumor blood vessels, allowing for the identification of tumor characteristics and assessment of tumor invasion. As a result, MRI is gradually replacing CT scans in the radiological evaluation of LPS (16, 17). In this particular case, the patient underwent abdominal CT or contrast-enhanced CT scans every 3 months for follow-up evaluations. This approach effectively tracks the recurrence and development of retroperitoneal tumors.

Surgical resection with negative margins is widely recognized the primary treatment for RPLPS (18). Studies have demonstrated that resection with clean margins under microscopy (R0 resection) leads to longer postoperative survival compared to resection with positive tumor margins under microscopy (R1 resection) (19). The scope of surgical resection for RPLPS remains controversial. Some studies suggest a method called ‘extended resection or septal resection’ to achieve radical resection. This involves removing adjacent organs and structures such as the kidney, colon, pancreas, spleen, psoas muscle, diaphragm, and retroperitoneal fat tissue vessels on the iliac side, even if they are not directly impacted by the tumor (20, 21). However, even with complete tumor removal, approximately 50% of patients still experience tumor recurrence within 5 years (22). For recurrent RPLPS, multiple reoperations may significantly improve long-term survival rates (23), although some studies suggest that an increase in recurrence and surgical frequency could lead to a higher recurrence rate (24). Our patient experienced 7 recurrences and underwent 8 complete resections. Remarkably, the patient’s survival period has reached nearly 8 years, which is exceptionally rare. The patient’s compliance with follow-up consultations has been exemplary, allowing for timely detection and treatment of each recurrence.

The efficacy of radiotherapy and chemotherapy in RPLPS remains controversial. According to a study by Littau MJ et al., adjuvant radiotherapy has been shown to improve survival rates in patients with tumors larger than 10 cm, but caution should be exercised when using it in patients with smaller tumors (25). Some studies have suggested that neoadjuvant radiotherapy (NART) combined with radical resection may result in better local control and prolonged survival compared to surgical resection alone. However, the long-term benefits of NART have not been thoroughly evaluated (26). As for adjuvant chemotherapy (AC) in RPLPS, anthracycline-based chemotherapy regimens, such as doxorubicin, are currently considered the first-line treatment for advanced or metastatic LPS (27). The combination of doxorubicin and ifosfamide appears to be more effective than doxorubicin alone, with doxorubicin showing greater benefit (28). However, a large phase III randomized controlled trial conducted by the European Organization for Research and Treatment of Cancer (EORTC) found that this combination regimen did not improve overall survival (OS) or recurrence rates (29). In conclusion, the effectiveness and long-term benefits of radiotherapy and chemotherapy for RPLPS still require higher-level evidence to be established.

Targeted therapy is currently a major focus of research in the treatment strategies for RPLPS. The amplification of MDM2 and the inhibition of p53 are recognized as key mechanisms contributing to the growth and progression of RPLPS. Therefore, targeting the MDM2-p53 axis has emerged as an appealing therapeutic approach (30). The first selective and potent MDM2 inhibitors discovered were Nutlins (Nutlin-1, -2, and -3), followed by RG7112, Idasanutlin, and SAR405838 (31). CDK4 is also identified as a potential therapeutic target for LPS. Zhang and his team have demonstrated that continued treatment with a CDK4 inhibitor (CDK4i) as a single agent leads to reduced proliferation of DDLPS cell lines and inhibits tumor growth in an *in vivo* xenograft model (32). Palbociclib, ribociclib, and abemaciclib are currently approved CDK4 inhibitors for clinical use, and they have shown promising results as single agents in the treatment of solid tumors (33). Anlotinib is an alternative treatment strategy for unresectable or advanced LPS, which has been shown to improve progression-free survival (PFS) and overall survival (OS) in patients with advanced STSs (34, 35). This patient was treated with anlotinib after experiencing a recurrence for the seventh time. However, the treatment did not yield positive results. Furthermore, ongoing investigations are exploring other therapeutic targets for retroperitoneal liposarcoma (RPLPS). Xu et al. conducted a study where they isolated and identified tumor-associated fibroblasts (TAFs) from retroperitoneal dedifferentiated liposarcoma (DDLPS). They discovered that the Tsp2 protein encoded by THBS2 promotes the formation of TAFs and tumor progression, suggesting that Tsp2 could be a significant component in the context of RPLPS and a promising therapeutic target for patients (36). Additionally, the research conducted by Yi et al. suggests that histone lysine N-methyltransferase 2D (KMT2D) is closely associated with the clinicopathological characteristics and unfavorable prognosis of gastric cancer, making it a potential biomarker for predicting the prognosis of gastric cancer (37). In our case, the comprehensive gene test results revealed a KMT2D mutation in the patient’s tumor. However, it remains to be determined whether this indicates a correlation between KMT2D and the poor prognosis of RPLPS, and whether KMT2D could serve as a novel therapeutic target for RPLPS. Further investigation is needed to verify these possibilities.

## 4 Conclusion

In summary, RPLPS is a rare malignant tumor with a high recurrence rate. CT and MRI are valuable auxiliary examination methods. Currently, surgery is the preferred treatment approach. The effectiveness of radiotherapy and chemotherapy in treating RPLPS has yet to be determined, but targeted therapy shows promise as a treatment strategy and a new avenue for future exploration. In cases of relapse after surgery, further surgical treatment may be considered, as multiple surgical resections have shown success in providing symptom relief. If complete removal of the tumor is not feasible, post-surgery options such as radiotherapy, chemotherapy, and targeted therapy can be utilized to achieve favorable outcomes. Regular monitoring, early detection, and prompt treatment are crucial in enhancing the quality of life and extending the survival time of patients with RPLPS. In this particular case, we will continue to monitor the patient closely and implement appropriate adjunctive treatments as needed to maximize the patient’s survival time.

## Data availability statement

The original contributions presented in the study are included in the article/**Supplementary Material**. Further inquiries can be directed to the corresponding author.

## Ethics statement

Ethical review and approval were not required for the study on human participants in accordance with the local legislation and institutional requirements. The patients/participants provided their written informed consent to participate in this study. Written informed consent was obtained from the individual(s) for the publication of any potentially identifiable images or data included in this article. Written informed consent was obtained from the participant/patient(s) for the publication of this case report.

## Author contributions

XW: Data curation, Formal analysis, Investigation, Writing – original draft, Writing – review & editing, Validation. XS: Formal analysis, Validation, Writing – review & editing. QS: Data curation, Formal analysis, Writing – review & editing. JW: Formal analysis, Validation, Writing – review & editing. JC: Data curation, Validation, Writing – review & editing.
