# Supplementary figures and images for: Recurrent retroperitoneal liposarcoma with multiple surgeries: a case report
Source: Front Oncol. 2024 May 3;14:1363055. doi: 10.3389/fonc.2024.1363055 (PMC11102049; doi:10.3389/fonc.2024.1363055)

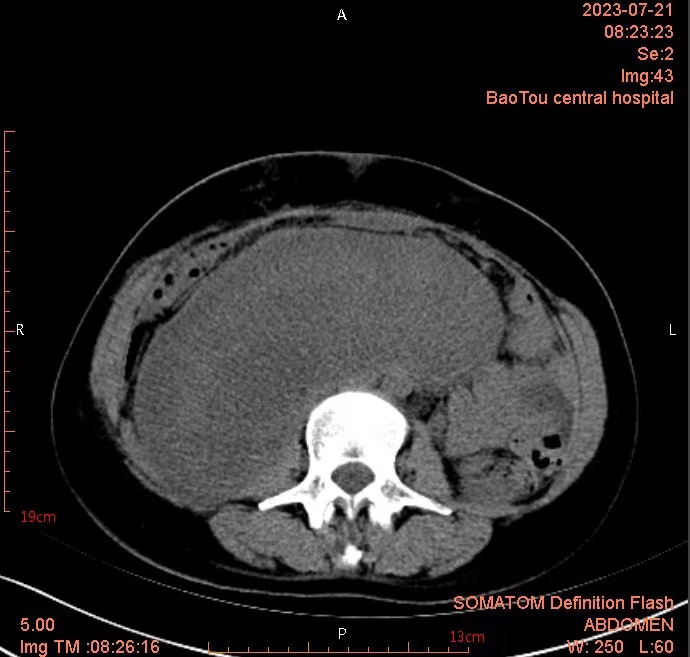

Supplement: Supplementary file 1 [file DataSheet_1.zip › Supplementary Material Presentation/1A.jpg]

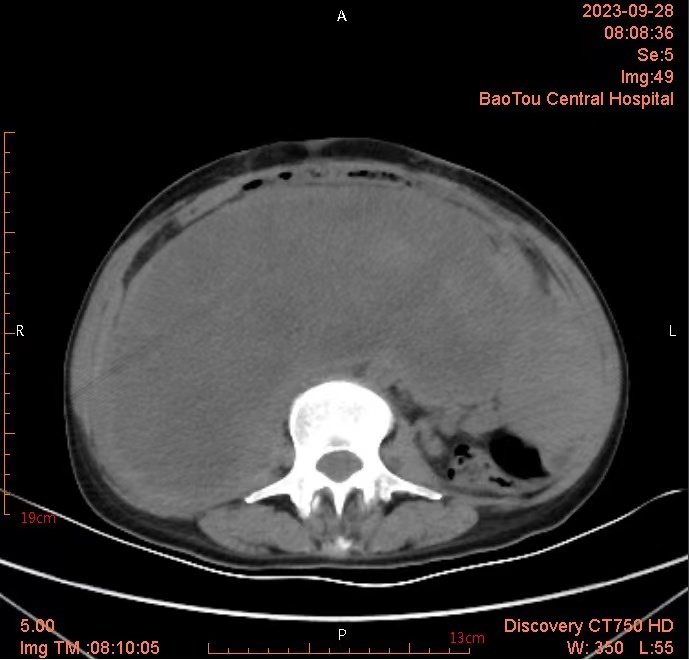

Supplement: Supplementary file 1 [file DataSheet_1.zip › Supplementary Material Presentation/1B.jpg]

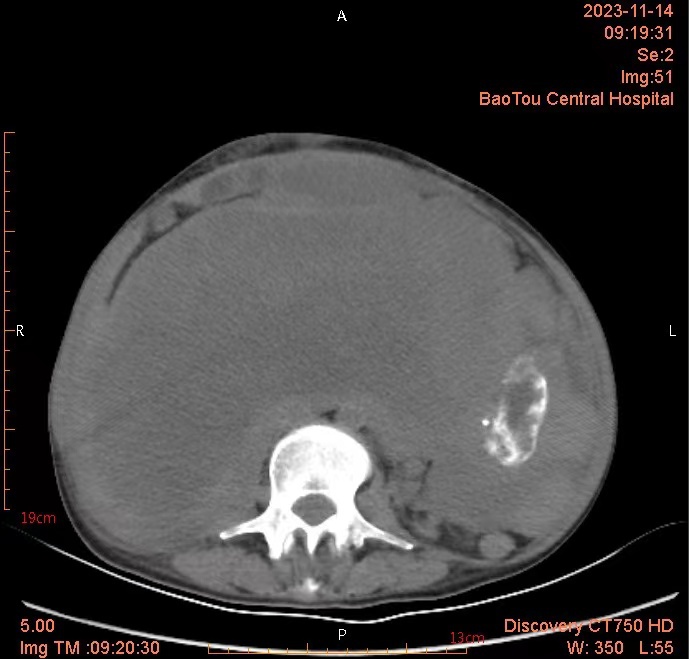

Supplement: Supplementary file 1 [file DataSheet_1.zip › Supplementary Material Presentation/1C.jpg]

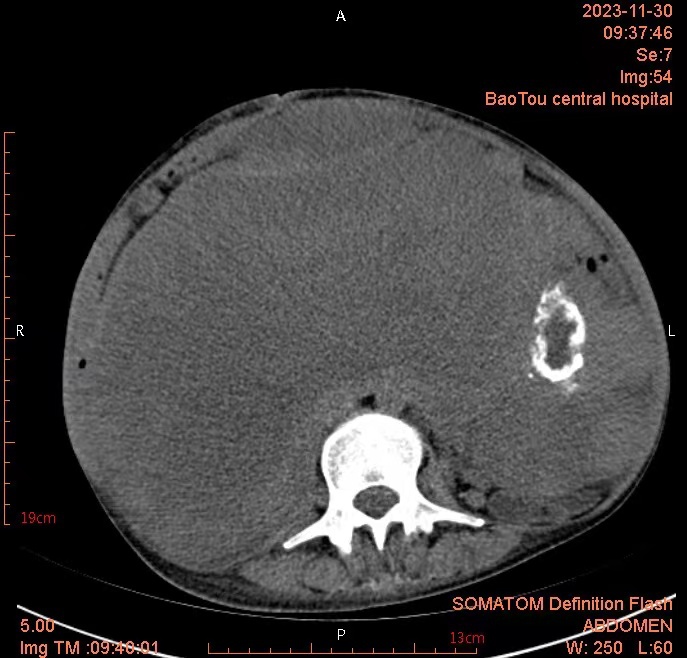

Supplement: Supplementary file 1 [file DataSheet_1.zip › Supplementary Material Presentation/1D.jpg]

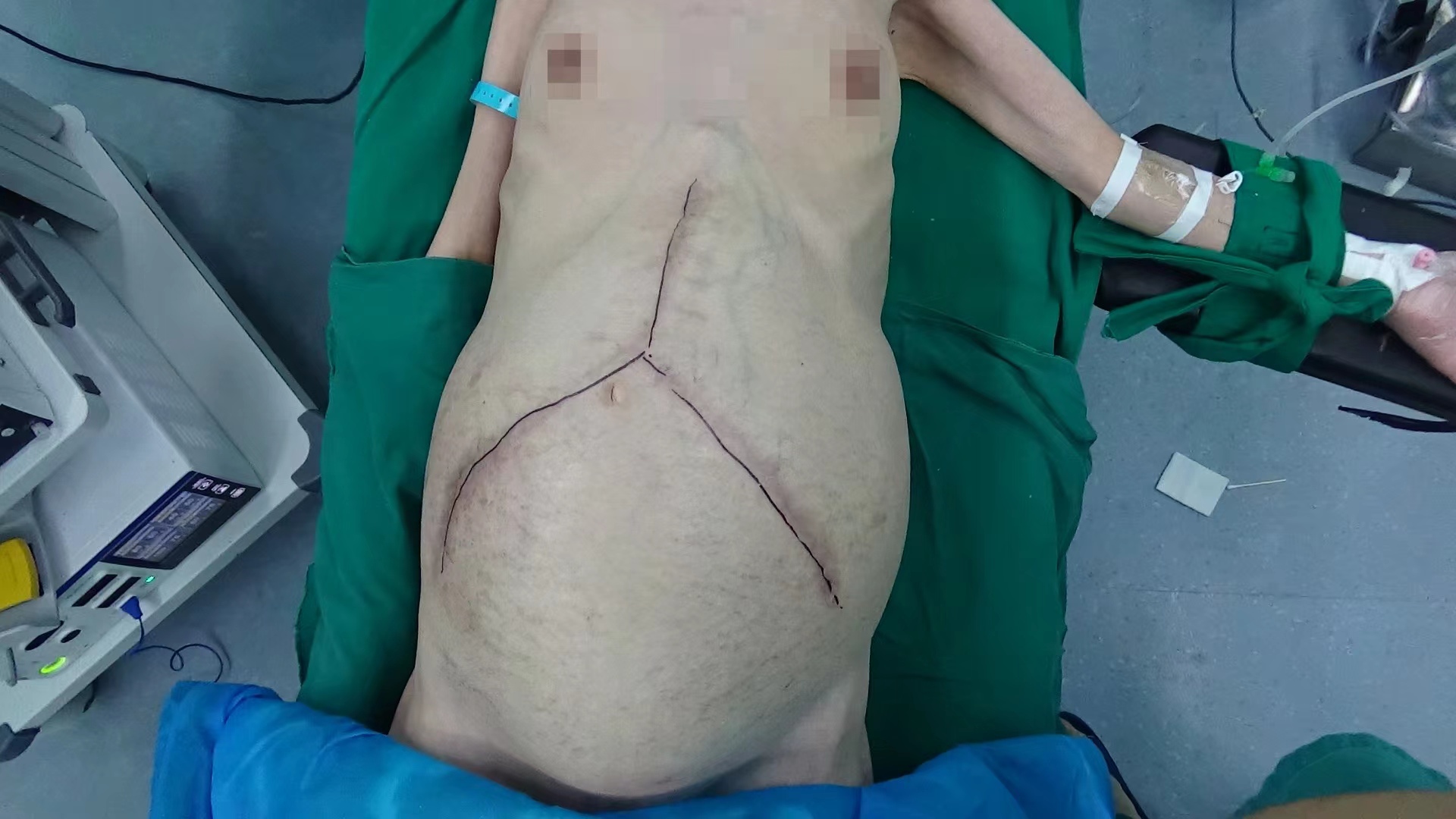

Supplement: Supplementary file 1 [file DataSheet_1.zip › Supplementary Material Presentation/2A.jpg]

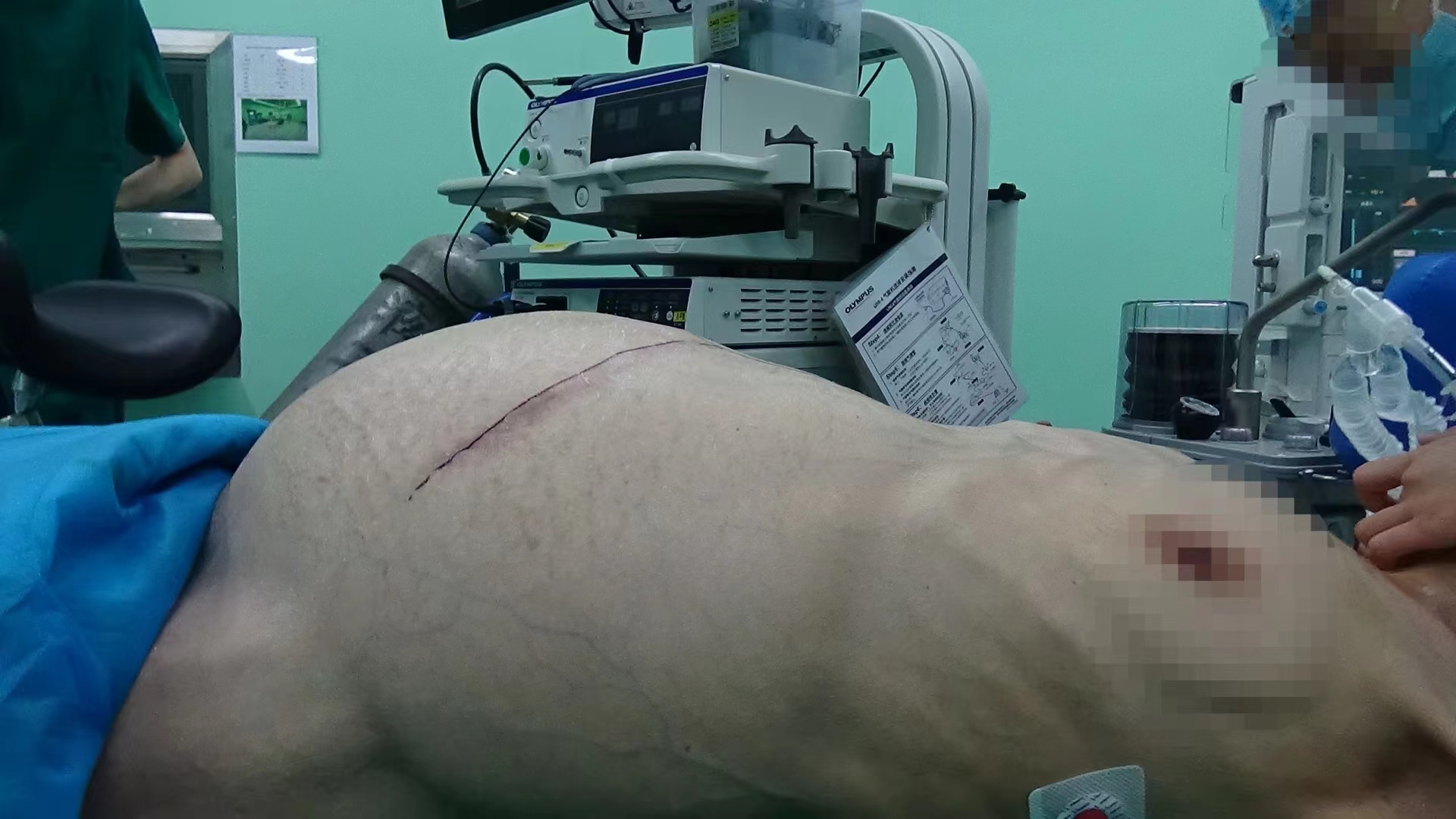

Supplement: Supplementary file 1 [file DataSheet_1.zip › Supplementary Material Presentation/2B.jpg]

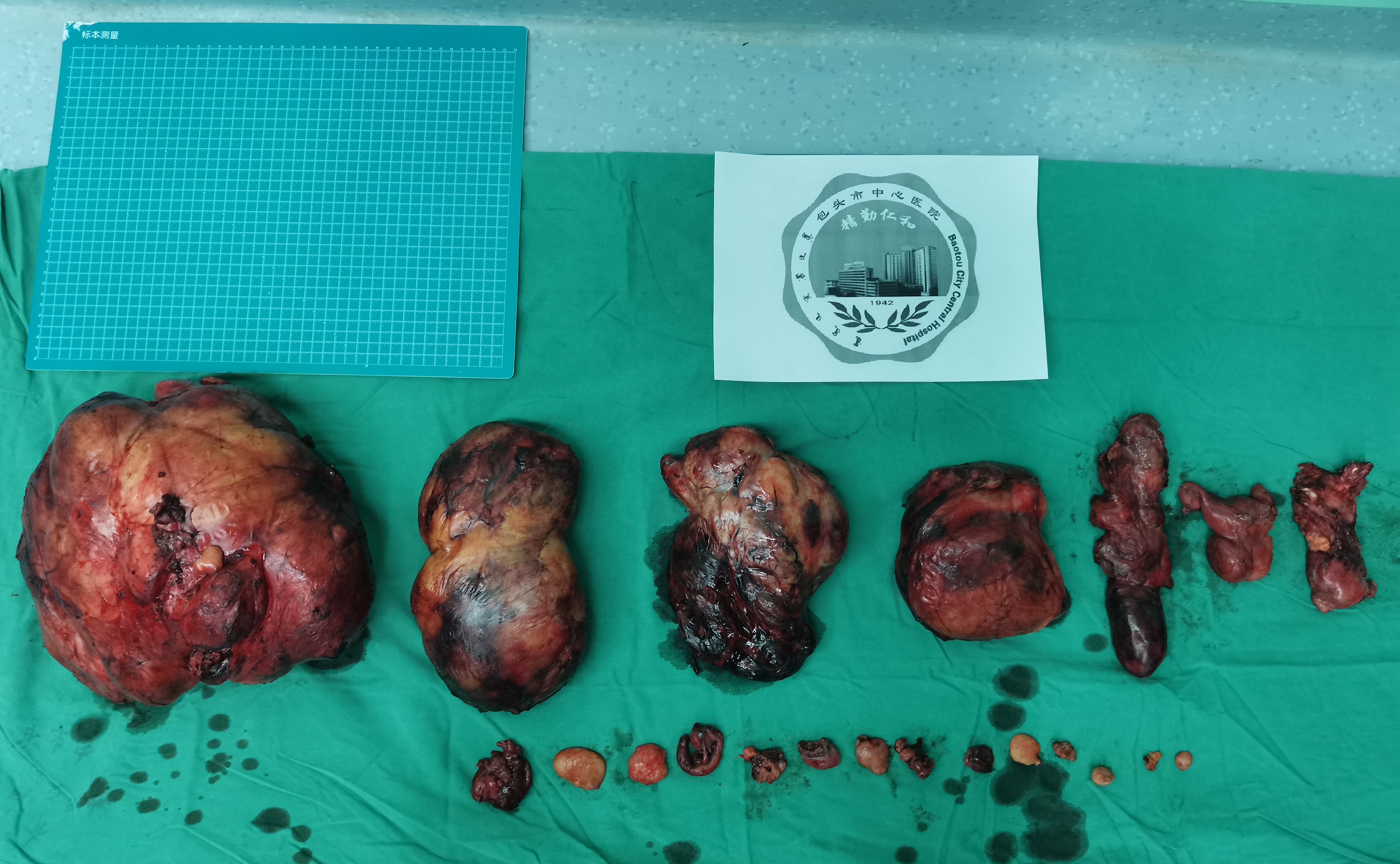

Supplement: Supplementary file 1 [file DataSheet_1.zip › Supplementary Material Presentation/2C.jpg]

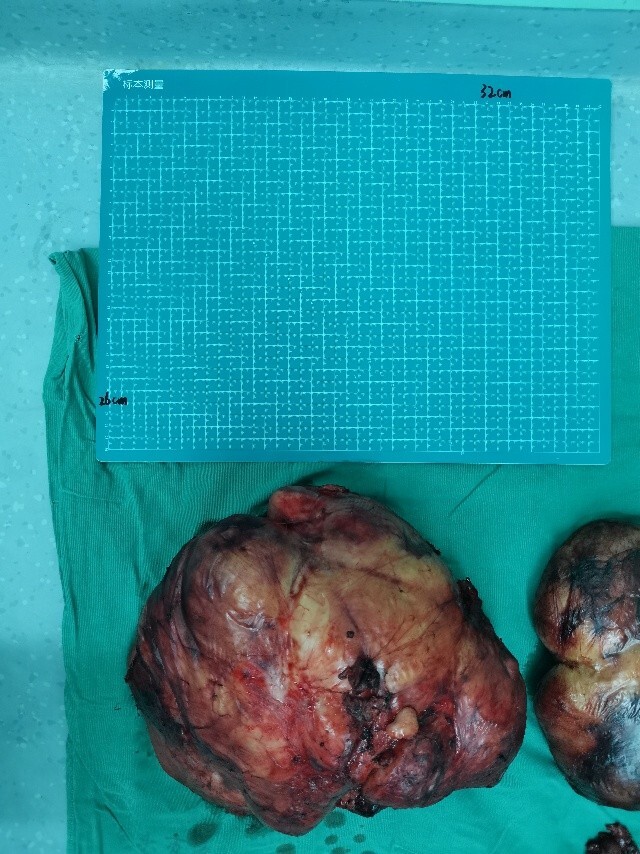

Supplement: Supplementary file 1 [file DataSheet_1.zip › Supplementary Material Presentation/2D.jpg]

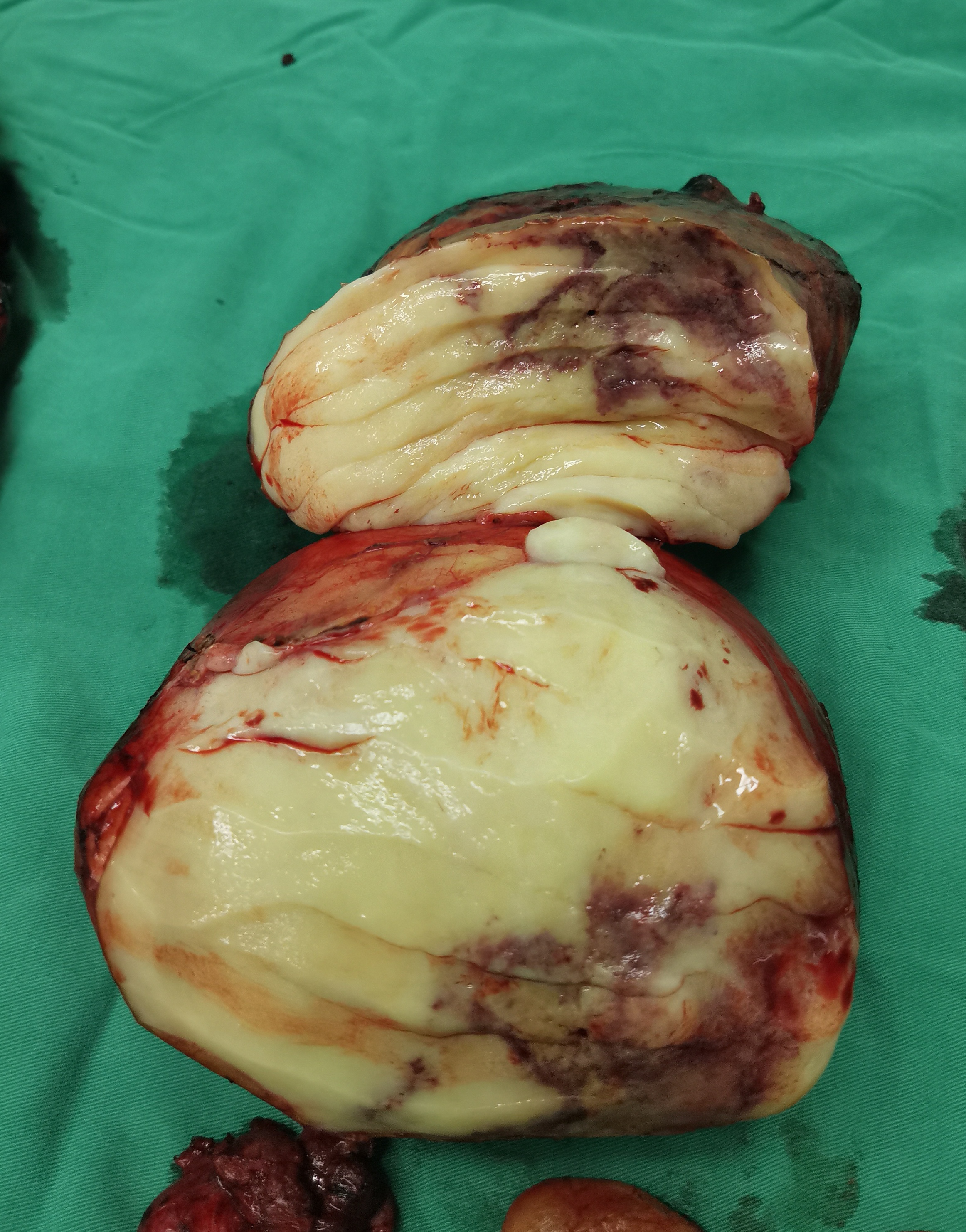

Supplement: Supplementary file 1 [file DataSheet_1.zip › Supplementary Material Presentation/2E.jpg]

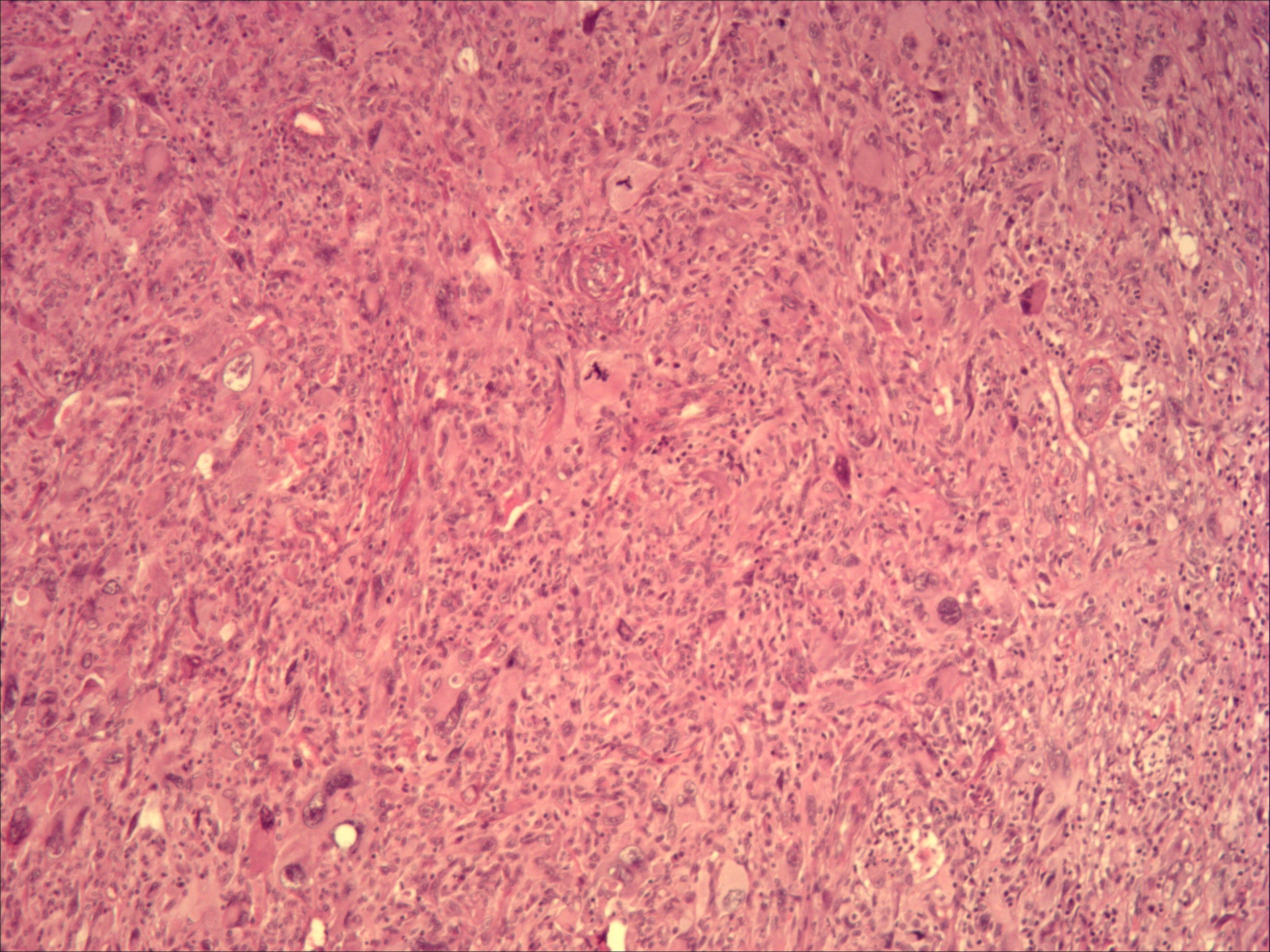

Supplement: Supplementary file 1 [file DataSheet_1.zip › Supplementary Material Presentation/3.jpg]

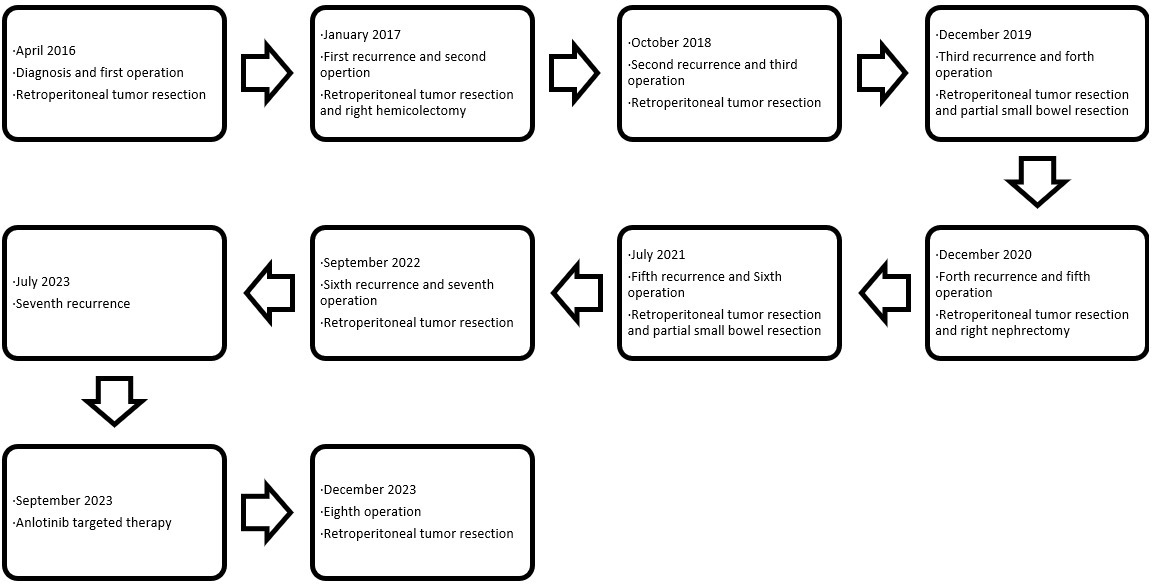

Supplement: Supplementary file 1 [file DataSheet_1.zip › Supplementary Material Presentation/4.jpg]
